# Sino-European Transcontinental Basic and Clinical High-Tech Acupuncture Studies—Part 4: “Fire of Life” Analysis of Heart Rate Variability during Acupuncture in Clinical Studies

**DOI:** 10.1155/2012/153480

**Published:** 2012-05-14

**Authors:** Gerhard Litscher, Lin-Peng Wang, Lu Wang, Cun-Zhi Liu, Xiao-Min Wang

**Affiliations:** ^1^Stronach Research Unit for Complementary and Integrative Laser Medicine, Research Unit of Biomedical Engineering in Anesthesia and Intensive Care Medicin, TCM Research Center Graz, Medical University of Graz, Auenbruggerplatz 29, 8036 Graz, Austria; ^2^Acupuncture and Moxibustion Center, Beijing Hospital of Traditional Chinese Medicine, Capital Medical University, Beijing 100010, China; ^3^Department of Neurobiology, Capital Medical University, Beijing 100069, China

## Abstract

This fourth part of a series of Sino-European high-tech acupuncture studies describes the first clinical transcontinental teleacupuncture measurements in two patients (cervical spine syndrome and tachycardia; both 27 years old) from the Beijing Hospital of Traditional Chinese Medicine affiliated to Capital Medical University, China. The electrocardiographic data were transferred to the Stronach Research Unit for Complementary and Integrative Laser Medicine and the TCM Research Center in Graz via conventional internet connections. Data analysis was performed in Graz using a new “Fire of Life” heart rate variability analysis. Analysis was performed without any technical problems in both subjects. Heart rate decreased significantly during acupuncture in the two patients from Beijing. At the same time, total HRV increased during acupuncture. The different influences of HRV (respiratory sinus arrhythmia, blood pressure waves, etc.) could be clearly documented using the new “Fire of Life” analysis.

## 1. Introduction

Recently, we performed several transcontinental acupuncture studies. Parts 1–3 of this series summarize some of our animal experimental and first clinical results, performed between institutions from Graz, Austria, Beijing, China, and Harbin, China [1–3]. Computer analysis of heart rate (HR) and heart rate variability (HRV) allows the identification of specific patterns in the fluctuations of the electrocardiogram (ECG) which reflects the effects of individual mechanisms involved in cardiovascular regulation. Based on the automatic assessment of these patterns, new scientific tools for evaluating the features of cardiovascular control have been developed [4, 5]. 

HRV has been investigated in normal subjects of various age groups and also in different cardiovascular diseases such as acute myocardial infarction, congestive heart failure, arterial hypertension, diabetes mellitus, and different autonomic dysfunctions [6, 7]. Beside HRV power spectral analysis, the so-called “Fire of Life” analysis (Huntleigh Healthcare, Cardiff, UK) is a new method of visualization of HRV, which has been described only in few scientific publications by our research group [8–12].

**Figure 1:**
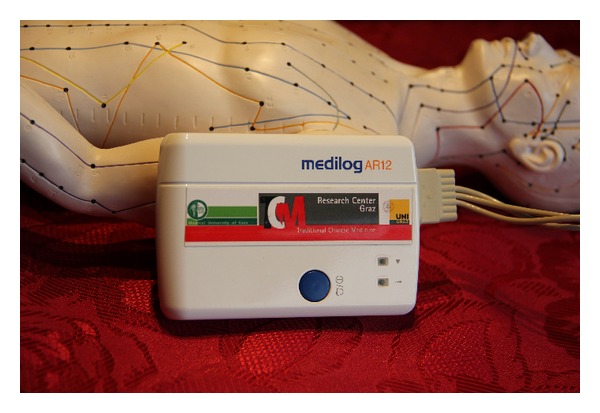
HRV equipment from Graz used for the clinical investigations at the Beijing Hospital of Traditional Chinese Medicine affiliated to Capital Medical University in China.

The aim of this study was to demonstrate the new “Fire of Life” HRV analysis in two patients from the Capital Medical University in Beijing. In both patients, the same type of monitoring equipment was used (Figure 1).

## 2. Materials and Methods

### 2.1. HRV Monitoring

An HRV medilog AR12 (Huntleigh Healthcare, Cardiff, UK, and Leupamed GmbH, Graz, Austria) system was used for cardiac monitoring in Beijing. The system is designed for a monitoring period of more than 24 hours. The sampling rate of the recorder is 4096 Hz. Therefore, R waves can be detected extremely accurately. All raw data are stored digitally on special memory cards. The data can be read by an appropriate card reader connected with a standard computer. The dimensions of the used HRV recorder are 70 × 100 × 22 millimeters, the weight is about 95 grams with batteries (compare Figure 1).

### 2.2. HRV Data Analysis

HRV is measured as the percentage changes in sequential chamber complexes (RR intervals) in the ECG. HRV can be quantified over time using registration of percentage changes in RR intervals in the time domain as well as the changes in the frequency range by analysis of electrocardiographic power spectra. Parameters are recommended by the task force of the European Society of Cardiology and the North American Society of Pacing and Electrophysiology [13]. Calculation of ECG power spectra is thought to provide an understanding of the effects of sympathetic and parasympathetic systems on HRV [1–7, 13]. Early work pointed out a few bands in the spectrum of HRV that could be interpreted as markers of physiological relevance. Associated mechanisms are thermoregulation which can be found in the very low-frequency band, blood pressure, and respiratory effects [13].

The new “Fire of Life” software analyzes HRV and displays it in a new way to help judge the function of the autonomic nervous system. Viewing this innovative kind of analysis can help to visualize how well the human body reacts to acupuncture. For offline inspection all ECG raw data can be displayed on a screen.

### 2.3. Patients

The investigations were performed in two patients (both female and both 27 years old) at the Beijing Hospital of Traditional Chinese Medicine affiliated to Capital Medical University. One of them (patient A) had a cervical spine syndrome and the other one (patient B) tachycardia. Both subjects were not taking any medication. The registration of the noninvasive parameters was in accordance with the Declaration of Helsinki of the World Medical Association.

### 2.4. Procedure

**Figure 2:**
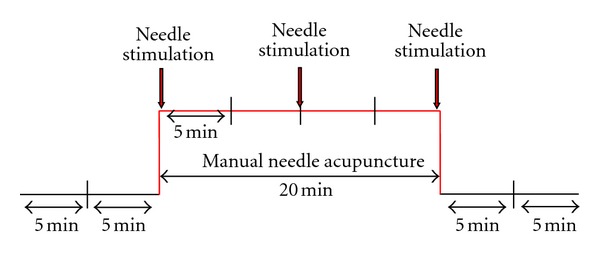
Recording profile. Each analysis segment consisted of 5 minutes. Altogether, a recording session of 40 minutes was performed in each patient.

The identical study design was used in both patients and included the following steps: three “Skintact Premier F-55” ECG electrodes (Leonhard Lang GmbH, Innsbruck, Austria) were fixed on the chest. The measurement procedure and the 5-minute segments (altogether 40 min) are shown in Figure 2.

### 2.5. Acupuncture Points

The following acupuncture points were used in the two patients: patient A (diagnosis: cervical spine syndrome) received manual needle acupuncture at Fengchi (GB20), Neiguan (PC6), and Tianzhu (UB10) and patient B (diagnosis: tachycardia) at Neiguan (PC6). For manual acupuncture stimulation, sterile single-use needles (length: 30 mm; diameter: 0.3 mm, Huan Qiu, Suzhou, China) were inserted perpendicularly to the skin at the respective acupoint(s). The needles were stimulated clockwise and counterclockwise for 15 seconds each, with two rotations per second, resulting in 30 rotations per stimulation. The stimulation was performed immediately after inserting the needle, 10 minutes later, and before removing the needle (see Figure 2).

## 3. Results

Data acquisition and data transfer over a distance of more than 7,600 km between China and Europe were performed without any technical problems.

### 3.1. Standard Analysis

**Figure 3:**
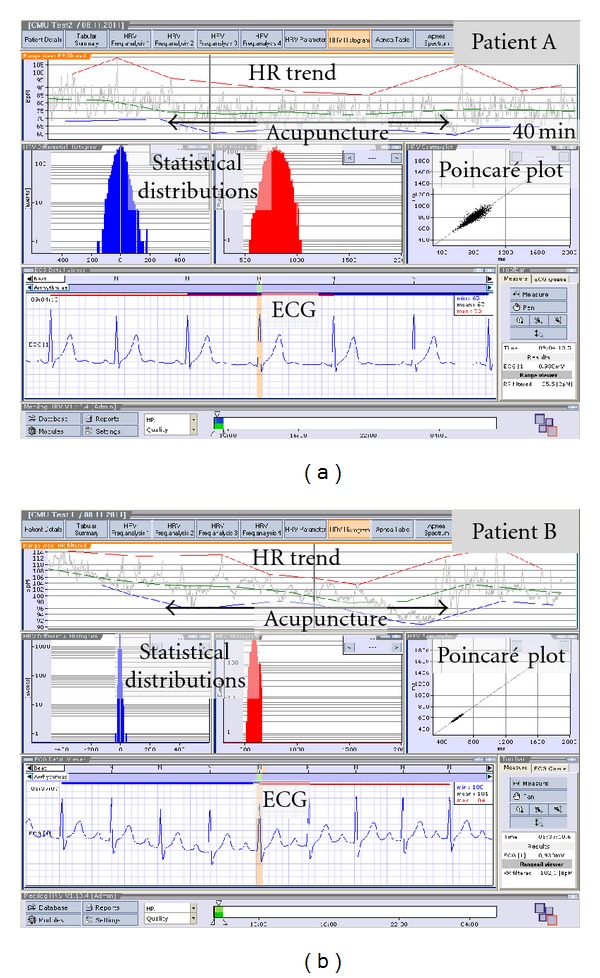
Data analysis of the ECG in the two patients (A and B). Note the decrease in HR in both patients during acupuncture.

Figure 3 shows the HR trends (upper panel), the statistical distribution of the RR intervals (middle panel, left and middle), the Poincaré plot (middle panel, right), and the raw ECG (lower panel) which was transferred from Beijing to the TCM Research Center in Graz.

The HR data from patient A over a period of 40 minutes are shown in Figure 3(a) (upper panel). At the beginning of the recording session, the mean HR was about 80/min. There are some minor artefacts during this period caused by movement. In the following acupuncture period, the patient was lying comfortably on a bed. The mean HR during this period was 70/min in patient A (Figure 3(a)) and about 100/min in patient B (Figure 3(b)). After finishing acupuncture, HR increased again slightly in both subjects (A: 75/min, B: 105/min).

### 3.2. HRV Scatterplots

**Figure 4:**
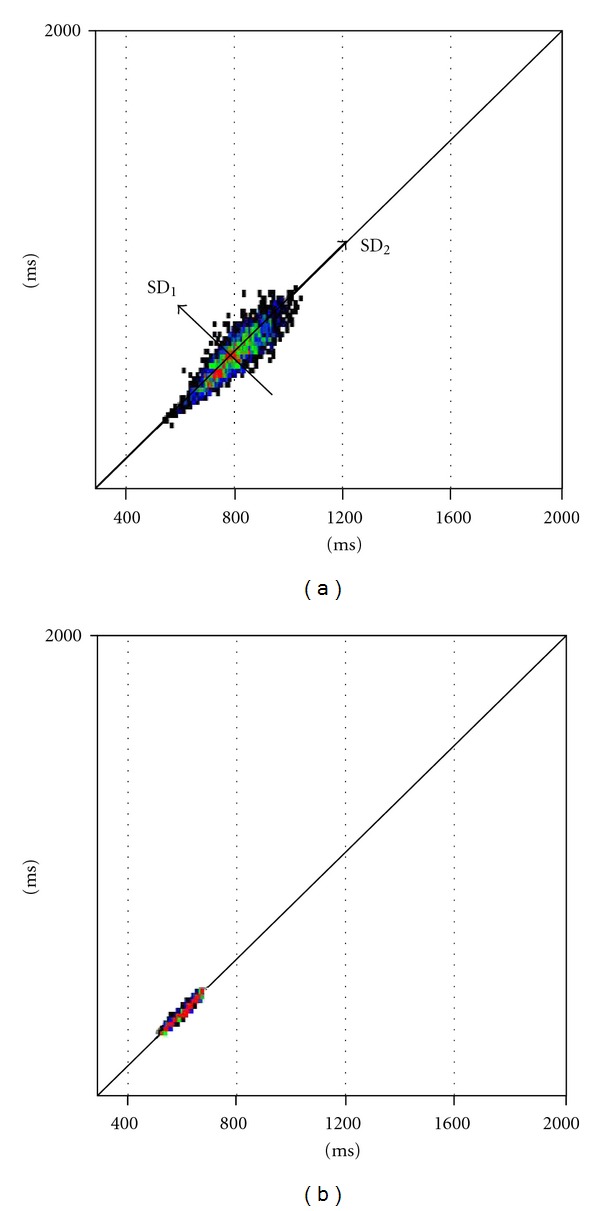
Quantitative beat-to-beat analysis of RR intervals (Poincaré plot). The results of patient A (a) are directly comparable (same scale) to those of patient B (b). Note the different shapes of the ellipses resulting in a different total heart rate variability.

The “Poincaré” plot is a technique taken from nonlinear dynamics [4, 8].  Figure 4 shows two Poincaré scattergrams in which each RR interval is plotted as a function of the previous RR interval. These graphical representations of cardiovascular dynamics result in elliptical types of shape (patient A). The ellipse is fitted onto the so-called “line of identity.” Standard deviation of the points perpendicular to the line of identity, denoted by SD_1_, describes short-term RR variability due to the respiratory component of HRV. The standard deviation along the line of identity, denoted by SD_2_, describes long-term variability [14]. In Figures 4(a) and 4(b) the two patients produced two ellipses of different shape and magnitude. Patient A (Figure 4(a)) showed a higher HRV associated with a big ellipse. Patient B (Figure 4(b)) produced an extremely reduced ellipse in which the RR points gravitate around the mean RR and the line of identity.

### 3.3. HRV Frequency Domain (“Fire of Life” Analysis)

**Figure 5:**
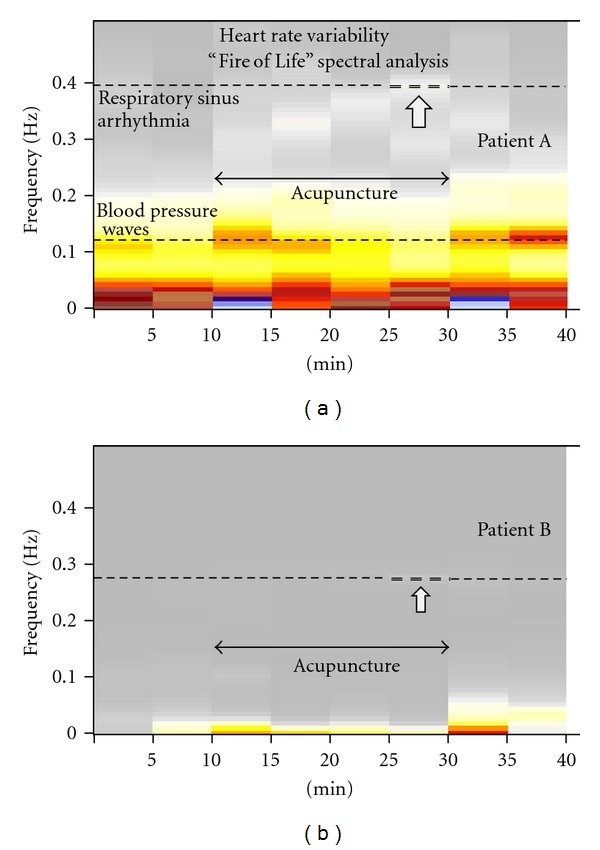
“Fire of Life” power frequency analyses. Heart rate variability (HRV) data of 40 minutes from patient A (a) and patient B (b) are shown.

The results of the “Fire of Life” HRV analysis of both patients are shown in Figure 5.

At the end of the acupuncture period (25–30 min), a small influence of respiratory sinus arrhythmia (frequency range 0.37–0.40 Hz) is recognizable in patient A (Figure 5(a)). This influence is much smaller in patient B (frequency range 0.28–0.30; Figure 5(b)). In addition the influence of blood pressure waves (frequency ~0.12 Hz) can be observed in patient A. The frequency range <0.05 Hz may also contain slowly changing effects from the renin angiotensin system and temperature regulation. However, the analysis and quantification of these latter parameters only make sense in long-term recordings. After acupuncture, during the last two resting periods (Figure 5(a)), the three main components of HRV analysis (respiratory sinus arrhythmia (very small), blood pressure influence, and thermoregulatory effects) are prominent in patient A. Similar effects, however extremely reduced, are shown in Figure 5(b) in patient B. Not only total variability (reduction of the “Fire of Life” in patient B) but also specific frequency component increases (e.g., influence from blood pressure waves and respiratory sinus arrhythmia) are noticeable. The neuromodulation of HRV is not as pronounced in patient B as in patient A. The “Fire of Life” burns much brighter in patient A than in patient B which can be seen in Figure 5 at the first glance.

## 4. Discussion

Transcontinental medical studies are rare [15–19]. Main fields of application are surgery [15], epidemiological assessments [16–18], and introducing new fields for academic health centers [19]. However, our research team was the first to perform “teleacupuncture” between Asia and Europe [10, 20, 21]. These first Sino-European transcontinental studies are at the moment mainly based on HR and HRV data acquisition and analysis using new modern methodological approaches like the “Fire of Life” analysis described in this fourth part of the series [1–3].

HRV is an index value of the neurocontrol of the heart. HRV can be quantified by simple calculation of the standard deviation of RR intervals of the cardiac cycles (total HRV) in the time domain [4, 13]. In addition, complex analyses of HRV in the frequency domain using different spectral analysis methods are possible [4, 13]. It is interpreted as a brainstem reflex with an afferent arc via the vagus and glossopharyngeal nerves and an efferent arc mainly via vagal fibres [22]. HRV has stochastic and rhythmic components. With spectral analysis variability can be classified into individual ranges which represent biological rhythms. The following influences can be distinguished for different ranges of HRV: (i) respiratory sinus arrhythmia (approximately 0.15–0.5 Hz); centrally nervous respiratory impulses and interaction with pulmonary afferents; (ii) the so-called “10-s-rhythm” (approximately 0.05–0.15 Hz); natural rhythm of cardiovascularly active neurons in the lower brainstem (circulatory center and its modulation by feedback with natural vasomotoric rhythms via baroreceptor feedback); (iii) longer wave HRV rhythms (approximately < 0.05 Hz); effects from the renin angiotensin system or temperature regulation as well as metabolic processes [4, 8, 13, 22, 23].

The scope of HRV is not yet completely clear, but it is known that there are intraindividual and interindividual variances, and that HRV depends on age, circadian variations (sleep-wake-cycle), physical condition, and mental and physical exertion. HRV can also be affected by diverse conditions such as age-related diseases (diabetic neuropathy, renal failure, essential hypertension, cardiac disorders, coronary artery disease, and intracranial lesions) and different medications [8]. The narrowness of HRV after heart transplantation [23] is similar to that seen in deep comatose patients and in brain-dead subjects [22] in whom complex reflex mechanisms are no longer generated or regulated in the brain. In contrast, heart transplantation totally interrupts peripheral autonomic afferences and efferences.

HRV can be used as reliable indicator of the state of health [4, 8, 21]. It becomes less random with the aging process and the appearance of age-related diseases. However, it has been demonstrated that in special syndromes like fatigue and stress, one can counteract this process using different preventive methods like sport [4, 8] or acupuncture [21, 24]. The latter has been demonstrated in recent investigations concerning patients with burnout syndrome as performed in a common teleacupuncture study between Beijing and Graz [21].

## 5. Conclusions

The following conclusions can be drawn from the results of the two patients of this preliminary study.

- Transcontinental data acquisition and analysis could be performed without any technical problems.
- Heart rate changes significantly during acupuncture of Fengchi, Neiguan, and Tianzhu in two patients from Beijing.
- Total HRV increases during acupuncture at the same acupoints as above.
- The different influences of HRV (respiratory sinus arrhythmia, blood pressure waves, etc.) can be clearly documented using the new “Fire of Life” analysis.
